# A modelling perspective on torque–frequency trade-offs in multifunctional lever systems driven by antagonist muscle pairs

**DOI:** 10.1242/jeb.250733

**Published:** 2026-01-30

**Authors:** Cas Jorissen, Sam van Wassenbergh

**Affiliations:** ^1^ Laboratory of Functional Morphology, University of Antwerp, Antwerpen 2610, Belgium; ^2^ Laboratory of Evolutionary Morphology of Vertebrates, University of Ghent, Ghent 9000, Belgium

**Keywords:** Biomechanics, Henneman's principle, Hill model

## Abstract

Rapid cyclic movements are generated by antagonistic muscle pairs contracting in an alternating pattern. The highest frequencies can be generated in balanced torque-producing systems with specialized muscle fibers. The system's frequency output is expected to change when it becomes more adapted to functions with conflicting mechanical demand, such as increased static torque production in one direction. This study first conceptualized how distinct factors (fiber type, muscle cross-sectional area, moment arm and inertial properties) could influence this torque–frequency trade-off. Special attention is given to Henneman's principle, as many of these systems contain both slow- and fast-twitch muscle fiber, typically organized in motor units, with the smallest, slow-twitch, fiber-rich motor units being recruited first. Next, we used Hill-type muscle models operating a Java sparrow's mandible as a case study for this framework. Our model showed that muscle fiber type strongly affects the frequency output, with a notable role for Henneman's effect causing the overdeveloped muscle to predominantly recruit slow-twitch muscle fibers. This leads to large muscle torque output overlap, which in turn reduces frequency. Once torque imbalance occurs, altering the other variables only slightly changes the frequency, suggesting a dominant role of muscle contractile properties. This means that the conflicting demands of multifunctional musculoskeletal lever systems such as bird beaks are also tightly linked to fiber type and motor unit roles such as endurance and precision of movement.

## Teaser

**Summary:** Low-inertia musculoskeletal lever systems are primarily constrained in their high-frequency movement output by fiber types and orderly recruitment, whereas mass, muscle cross-sectional area and moment arms have a minimal effect.

## INTRODUCTION

The evolution of musculoskeletal systems with a single, dedicated function can lead to extreme performances. For example, astonishingly high frequencies of cyclical movement (up to 1000 Hz) are generated in the sound-producing organs and flight muscles of insects (Gau et al., 2023). To achieve this feat, these animals use asynchronous muscles, meaning that the action potentials are decoupled from the muscle's activation. High-frequency movements become more difficult to achieve when each contraction is related with an action potential, as in synchronous muscles (for a comprehensive review, see Mendoza et al., 2023). The fastest known contractions of this muscle type (up to 250 Hz) are generated in sound-producing organs of songbirds (Elemans et al., 2008), bats (Elemans et al., 2011) and toadfish (Young and Rome, 2001) by ‘superfast’ muscles (for an in-depth review, see Rome and Lindstedt, 1998). Although these systems reach extremely high frequencies, adaptations required for extremely fast activation and relaxation of muscle to produce an extremely short force pulse (e.g. high fractions of non-contractile elements such as sarcoplasmic reticulum in the muscle) inevitably compromise these systems in generating power (Rome and Lindstedt, 1998). However, many examples of specialized musculoskeletal systems for high power output exist: hindlegs of lizards with high accelerations during sprinting (Curtin et al., 2005), frogs hindlegs during jumping (Lutz and Rome, 1994), wings of birds during fast take-off (Askew and Marsh, 2001) or powerful head elevation in suction-feeding fish (Li et al., 2022). Power output can be increased further when muscles work in tandem with elastic springs and latches in systems such as ballistically projecting tongues in chameleons (De Groot and Van Leeuwen, 2004) or salamanders (Deban et al., 2007), legs of jumping locusts (Bennet-Clark, 1975) or heads of pivot-feeding seahorses (Van Wassenbergh et al., 2009).

In contrast to such systems with specialized tasks, whenever multiple functions need to be fulfilled by a single musculoskeletal system, performance optimization of the different functions will be compromised (e.g. Van Damme et al., 2002; Garland et al., 2022). As the complexity and variety of behaviors of animals often calls for shared functions of body parts, trade-offs are often inevitable because of functional conflicts (Garland et al., 2022). For example, increased endurance requires a shift to more aerobic metabolism in muscles, which results in a decrease in power and maximal frequency output due to a reduction in the myofibrils to make space for an increase in mitochondria (Rome and Lindstedt, 1998). This is illustrated by the tail shaker of rattlesnakes, which sustains shake frequencies of up to 90 Hz for hours but cannot achieve the top frequencies of other systems containing superfast muscles because of the intrinsic endurance capacity of these rattlesnake muscles (Chadwick and Rahn, 1954; Schaeffer et al., 1996). Understanding the biomechanics involved in such trade-offs is not only essential for understanding how complex integrated systems work but is also central to the field of evolutionary biology (e.g. Garland and Carter, 1994).

A trade-off that has received considerable attention in biomechanical works (Alcazar et al., 2019; Biewener et al., 2014; Bottinelli et al., 1991; Close, 1964; Edman, 1988; Ranatunga and Thomas, 1990; Labonte, 2023), and subsequently is regularly adopted in functional and evolutionary biology (e.g. Westneat, 1994; Herrel et al., 2009), is the force–velocity trade-off in muscle-operated lever systems. The force–velocity trade-off theory states that lever displacements with larger forces at the output point on the lever happen at lower velocities at that point. Similarly to shifting gears on a bike, modifying the geometry of the lever system will determine how the power output of a muscle results in either a higher torque output at low velocity (i.e. ‘low’ gear) or a low torque output at high velocity (i.e. ‘high’ gear). However, force and velocity are not in trade-off with each other in Newton's second law of motion, as higher force means higher acceleration of a given mass, which over time can result in higher velocities. Studies have concluded that the applicability of the classical force–velocity trade-off of lever systems to animal motions depends on the specific dynamics of the action considered (Labonte, 2023; Polet and Labonte, 2024; McHenry, 2011; Van Wassenbergh et al., 2005).

The biomechanics of muscle-driven lever systems are sometimes fundamentally different from that of a single muscle pulling on a lever. To perform repetitive back-and-forth movements, musculoskeletal levers are often operated by antagonistic muscle pairs that alternatingly move the lever in opposite directions. Examples include locomotory systems (running, flying, digging, etc.) and feeding apparatus (chewing, manipulating food). The performance in terms of maximum cycle frequency and amplitude of the lever will depend on the properties of both muscles of the antagonist pair and how they are activated. The time it takes for the antagonist muscle to relax will influence the ideal activation timing of the agonist. Hence, the evolution of the properties of one of the muscles will have consequences for the way the muscle pair will work together for the repetitive movement. Here, we will focus on trade-offs between two functions: (1) exerting a high torque by the lever in one of the directions in a static situation, and (2) executing high-frequency movement. We refer to this as the torque–frequency trade-off.

The goals of our study are to: (1) provide a conceptual framework of how distinct factors are expected to influence the torque-frequency trade-off; and (2) apply this framework to evaluate an important model system in evolutionary biology, namely the trade-off between static bite force (or mandible torque output in the beak-closing direction, important for breaking hard seeds) and high-frequency movement of the beak during feeding (food manipulation before and after seed crushing) and singing in songbirds. Darwin's finches are well known for their adaptive radiation in the Galapagos, where species specialized on different seeds with a variety of sizes and hardness. Several species of this group produce complex songs with high trill rates and associated high-frequency open–close movements of the beak to attract potential mates (Grant and Grant, 1997, 1998). A trade-off between static bite force and high-frequency movement is present in the beak of finches: species with larger beaks, which can generate and withstand higher bite forces, sing using slower beak rates than birds with small beaks (Herrel et al., 2005, 2009; Podos, 2001). However, how adaptations for larger bite forces influence the torque–frequency trade-off is still unclear.

List of symbols and abbreviations

*A*: activation level
AAD: antagonistic activation delay
*A*_max_: maximal activation multiplied by the proportion of motor units activated
*c*: residual force factor of the force–length curve
*C*: small constant because the activation cannot be zero
*F*_be_: force output of the buffer element
*F*_ce_: force output of the contractile element
*f*_l_(*l*_ce_): force–length relationship
*F*_m_: muscle force output
*F*_max_: maximal isometric force output
*F*_pee_: force output of the parallel elastic element
*F*_see_: force output of the serial elastic element
*f*_see_: non-linear serial elastic element function
FT: fast-twitch
*f*_v_(*v*_ce_): force–velocity relationship
*K*: constant describing the curve of the force–velocity relationship
*l*_ce_: length of the contractile element
*l*_min_: rest length of the buffer element
*l*_opt_: muscle length for highest isometric force output
*l*_see_: length of the serial elastic element
*l*_slack_: rest length of the tendon
MU: motor unit
*N*: eccentric force multiplication
ST: slow-twitch
*T*_hr_: half relaxation time, time from the maximal force output to *F*_max_/2
*T*_imp_: time between the start of two successive pulses
*T*_ttp_: time to peak, duration between start force production and when it reaches maximal force
*v*_ce_: contractile velocity of the contractile element
*v*_max_: maximal contractile velocity
*w*: relative width of the force–length curve
ε: strain
ε_be_: reference compression
ε_pee_: reference strain of the parallel elastic element
ε_ref_: reference strain

## CONCEPTUAL FRAMEWORK

**Fig. 1.**
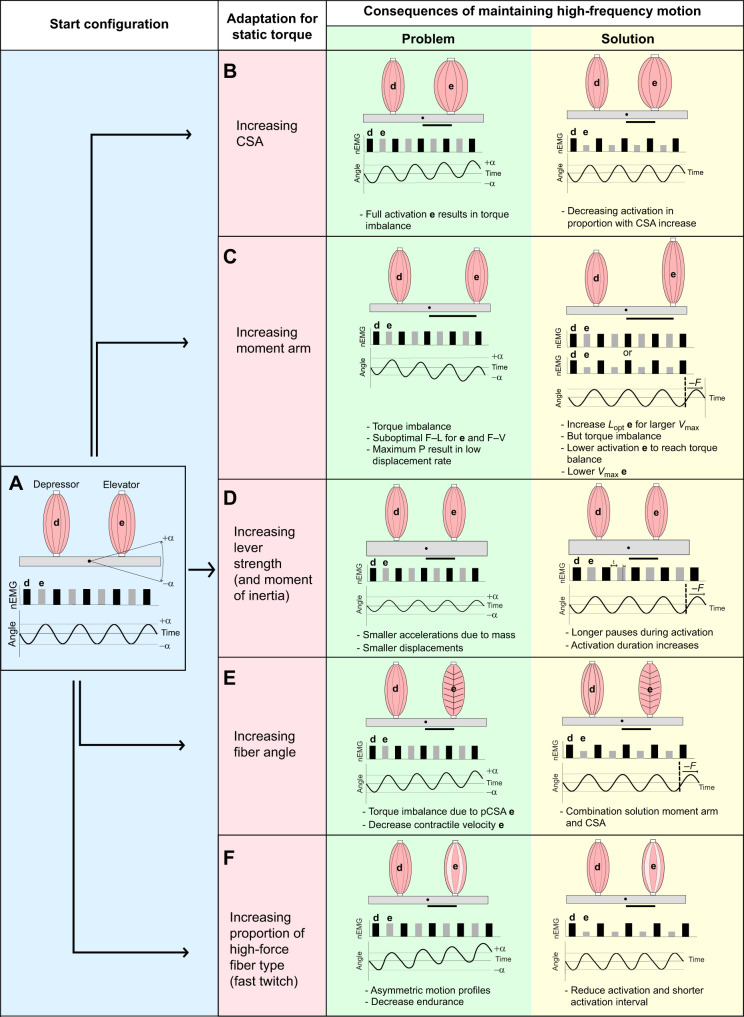
**Schematic overview of the conceptual framework.** With (A) the standard configuration, (B) an increased cross-sectional area (CSA) of the elevator muscle, (C) an enlarged moment arm of the elevator muscle, (D) an increased fiber angle of the elevator muscle, (E) an increased mass of the lever and (F) when the muscles contain both fast-twitch and slow-twitch muscle fibers. The normalized EMG (nEMG) indicates the activation levels that generate the movements shown. The arrow and *F* indicate that the frequency of the movement decreases. The black vertical bar underneath the levers shows the moment arm of the elevator muscle. F–L, force–length; F–V, force–velocity.

This section aims to theoretically describe the influences of adaptations for increased static torque production in one direction on frequency. In this thought experiment, we start by describing the simplest form of a generic antagonistic muscle lever system (see Fig. 1A): a lever with the joint placed in the center, and right in between an antagonistic muscle pair with identical intrinsic properties (e.g. same maximal isometric force *F*_max_ and maximal contractile velocity *V*_max_) and parallel fibers. We assume that the muscles' contractile regime (e.g. in terms of power production) is optimized for this geometry to rotate the lever up (elevation to angle +α, or the ‘elevated position’) and down (depression to angle –α, or the ‘depressed position’) in a cyclical pattern at a constant amplitude of 2α. Next, adaptations for larger torque production are applied to the elevator muscles. For simplicity, we will ignore external forces such as gravity or joint friction and assume that the muscles are massless and tendons have no influence on the dynamics.

### Start model

In this thought experiment, this baseline model is adapted to produce the highest possible frequencies within the limits of muscular performance. Owing to the equality in muscle properties and the symmetrical geometry, the antagonistic torque and power productions of the muscles are in balance. Therefore, activating both muscles fully out of phase results in the highest frequencies (Fig. 1A).

### Cross-sectional area

An increase in cross-sectional area (CSA) of the elevator muscle creates a torque production imbalance between the muscles if the muscle activation intensity is maintained (Fig. 1B). If, for example, the elevator muscle shifts to a CSA that is double that of the depressor, the elevator's torque will become twice that of the depressor and so will the muscle's intrinsic capacity to produce power. This will result in higher upward accelerations and velocities of the lever and will shift the lever's mean position further in the elevator (+α) direction with each cycle. The solution to reach torque and power output balance is to only activate the elevator muscle submaximally, with the level of activation in inverse proportion to the increase in CSA. This will bring the mechanical output back to that of the start model, and hence, the frequency can stay equal.

### Moment arm

An increase in the moment arm of the elevator muscle also introduces a torque imbalance between the antagonist muscles (Fig. 1C). At first glance, the effect of increasing moment is similar to that of an increase in CSA. Indeed, when doubling the moment arm, the torque increases similarly. When the moment arm increases, the fibers of the elevator muscle will be stretched more when the lever is displaced to the depressed position. Then, to reach the elevated position, the elevator muscle needs to contract more to reach the elevated position. Consequently, the elevator's dynamic force output will be different due to both the force–length and force–velocity relationships. Given that our start configuration was assumed to operate optimally along the muscle's force–velocity relationship (i.e. it contracts at the speed for maximal net power output over the cycle), the duration of the upward displacement to reach the elevated position (+α) increases. In turn, the depressor muscle will be activated too early in the elevation phase to reach the elevated position, and this will result in smaller displacements in the elevation (+α) direction. This results in a skewed movement to the depressed direction. How the combined effects of the elevator's increased torque at low contraction speeds and sub-optimal gearing for power output influence the kinematic pattern is difficult to predict, but a negative effect on frequency is inevitable.

What modifications to the elevator muscle could bring such a system back in dynamical balance? A first important parameter to consider is the length of the elevator muscle. By increasing the length, the strain rate of the muscle (i.e. relative contraction speed in muscle lengths s^−1^) can be maintained in response to the longer absolute distance over which the muscle has to shorten. Maintaining full activation of both elevator and depressor muscles would result in higher elevator torques and powers, so that also a reduced level of activation is required to regain a dynamically balanced, cyclical motion at the original frequency. By only decreasing the activation intensity (i.e. without changing muscle length), the velocity of elevation will reduce, again introducing a velocity imbalance between the antagonists. Spacing the activation signals further apart in time, which decreases the torque overlap (i.e. simultaneous elevation and depression torques of which the effect on movement is cancelled out), might be beneficial but logically results in a frequency reduction.

### Mass

Larger torques during biting increase the risk of fracturing the lever. Adaptations for preserving (or increasing) the safety factor of the skeletal element under higher bite loading include producing a tougher lever. To achieve this, the skeletal element's material density or thickness must increase and, hence, the mass increases. The influences of muscle and inertial properties on muscle-driven motion have recently been described in detail by Labonte (2023). If a muscle contracts against a sufficiently small mass, the acceleration is almost instantaneous, meaning that the *V*_max_ is quickly reached and, therefore, *V*_max_ is the constraining factor. With the assumption that the muscles are always fully activated and produce maximal force, the increase in mass will inevitably lead to reduced accelerations and decelerations (Fig. 1E). As long as *V*_max_ is reached relatively quickly in the acceleration phase, changing mass might have a relatively small effect on frequency. When muscles work at contraction speeds to deliver optimal power output before the increase in lever inertia, sub-optimal power output at lower speeds will also reduce movement speed. Increasing the lever mass in our start configuration will result in rotations smaller than the required movement α. To solve the reduced amplitude issue, both muscles should be kept fully activated but the delay between the antagonist activation increases (and duration may also slightly increase). This can result in movements keeping the original rotation of 2α, but this will inevitably be at lower frequencies.

### Muscle architecture

The architecture of the muscles can have a strong functional influence. Parallel muscles consist of fibers that extend along almost the entire length of the muscle. With many sarcomeres in series, the muscle can shorten at high absolute velocities (Bodine et al., 1982; Gans, 1982). In contrast, pennate muscles consist of fibers that are shorter and are oriented at an angle to the muscles' axis of force production. Pennate muscles allow more sarcomeres in parallel, therefore increasing the physiological CSA of the muscle, implying increased static force generation capacity (Powell et al., 1984). An important descriptor of the mechanical consequence of variation in muscle architecture is the ratio of muscle velocity to fiber velocity: the architectural gear ratio (Eng et al., 2018). This ratio is a function of pennation angle, but also depends on the dynamics of the muscle contraction as this ratio decreases as the muscle contracts while generating more force (Azizi et al., 2008).

The effect of increased pennation angle (or other source of architectural gear ratio decrease) on frequency output can be seen as a combination of the above-described influences from increasing CSA and moment arm (Fig. 1D). The torque production capabilities of the muscle improve due to the pennation, but the maximum contractile velocity of the muscle as a whole decreases. Hence, reduced activation would reduce both torque productions and contractile accelerations near the onset of movement, and with shorter muscle fibers the maximal shortening speed of the muscle reduces. Because of a shift towards a pennated muscle, the movement will become slower, introducing an imbalance in the lever movements and, hence, reducing the maximally achievable frequency.

### Muscle fiber type

Muscle fiber type will affect torque and frequency in different ways. We will distinguish between slow-twitch (ST) and fast-twitch (FT) muscle fibers. FT muscle fibers have a faster force development and relaxation, and higher *V*_max_ and *F*_max_. Changing ST fibers to the FT fiber variant for the elevator muscle will thus lead to both increased static torque capacity and increased frequencies due to the elevator fiber's increased maximal speed and power (Fig. 1F). The faster force rise and relaxation times will reduce the antagonistic torque overlap so that opposing torques can follow each other quickly. In case the hypothetical change results in a higher proportion of FT fibers in the elevator muscles compared with the depressors, again, reduced activation intensity of the elevator muscles should occur to achieve a balanced, cyclical motion. This will limit the potential improvement in the maximal frequency output of the system. Still, there is no trade-off, as the FT fibers are better for both high torques and high frequency in our lever system. However, there will be an important trade-off between endurance capacity on the one hand, and both torque and frequency on the other hand.

**Fig. 2.**
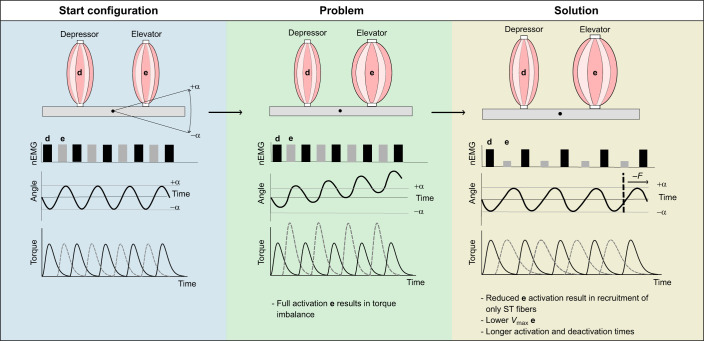
**The combined effect of having two types of muscle fibers and an increase in CSA of the elevator muscle.** The nEMG shows the normalized activation that generates the muscle torques and results in the movements shown in degrees. ST, slow twitch.

The musculoskeletal mechanics of our lever systems will become more complex if there is both imbalance in the lever systems' torque, displacement or velocity discussed in the examples above, and a mixture of ST and FT fibers present in the muscles. Henneman's size principle, or the principle of orderly recruitment for motor unit usage (Henneman, 1957; Henneman and Olson, 1965), states that the smaller motor units (MUs) (i.e. MUs with smaller motoneuron size which produce low force) are recruited before the larger MUs (i.e. MUs with large motoneuron sizes which produce high force). Smaller MUs of mixed fiber-type muscles consist mostly of ST fibers, whereas larger MUs consist predominantly of FT fibers (Henneman et al., 1965a,b). Hence, an increased proportion of ST fibers will be recruited when the muscle activation level and force output are decreased. Note that this situation in which a reduced force output is generated was often the only possibility to achieve a balanced cyclical motion in geometrically asymmetric musculoskeletal lever systems. Consequently, torque imbalances between antagonistic muscle pairs (Fig. 1B–E) may cause a shift towards the recruitment of slower fiber types. This effect can amplify the strength of the torque–frequency trade-offs caused by the various adaptations described above (Fig. 2).

### Conclusions

This thought experiment suggests that adaptations for larger torque production in one of the muscles of an antagonistic muscle pair often results in the system's frequency reduction. Although the frequency decreases, the production of similar oscillatory movements is still possible by reducing the muscle activation intensity of the ‘stronger’ muscle. For systems composed of muscles with a mixture of ST and FT muscle fibers, decreasing the intensity of muscle activation might result in the predominant activation of ST muscle fibers, further altering the antagonistic balance and, therefore, frequency. The combination of different muscle fibers in an imbalanced torque producing musculoskeletal system might amplify the strength of the torque–frequency trade-offs.

We investigated this generalized conceptual framework (Figs 1 and 2) using a forward dynamics computational model based on the anatomy of a songbird *Lonchura oryzivora* (Java sparrow). Using this model, we evaluated how the variables discussed above affect the frequency output in multifunctional lever systems. Specific attention was paid to the hypothesis of how the required level of muscle activation may interfere with the recruitment of fiber types with different contractile properties (Henneman's effect; Fig. 2).

## MATERIALS AND METHODS

### Java sparrow beak characterization

To set up a biologically realistic case study, the jaw system of the Java sparrow [*Lonchura oryzivora* (Linnaeus 1758)] was chosen. This estrildid finch species has an above-average bite force (Van Der Meij and Bout, 2004), and shows a clear torque imbalance between the muscles that open and close the beak (Genbrugge et al., 2011, Fig. 3C). Several variables of the musculoskeletal system involved in mandible movement were measured to serve as input in our computational model (see below). Note, however, that it was not our intention to model this system as accurately as possible but to use a model that is sufficiently realistic to represent a songbird beak system.

**Fig. 3.**
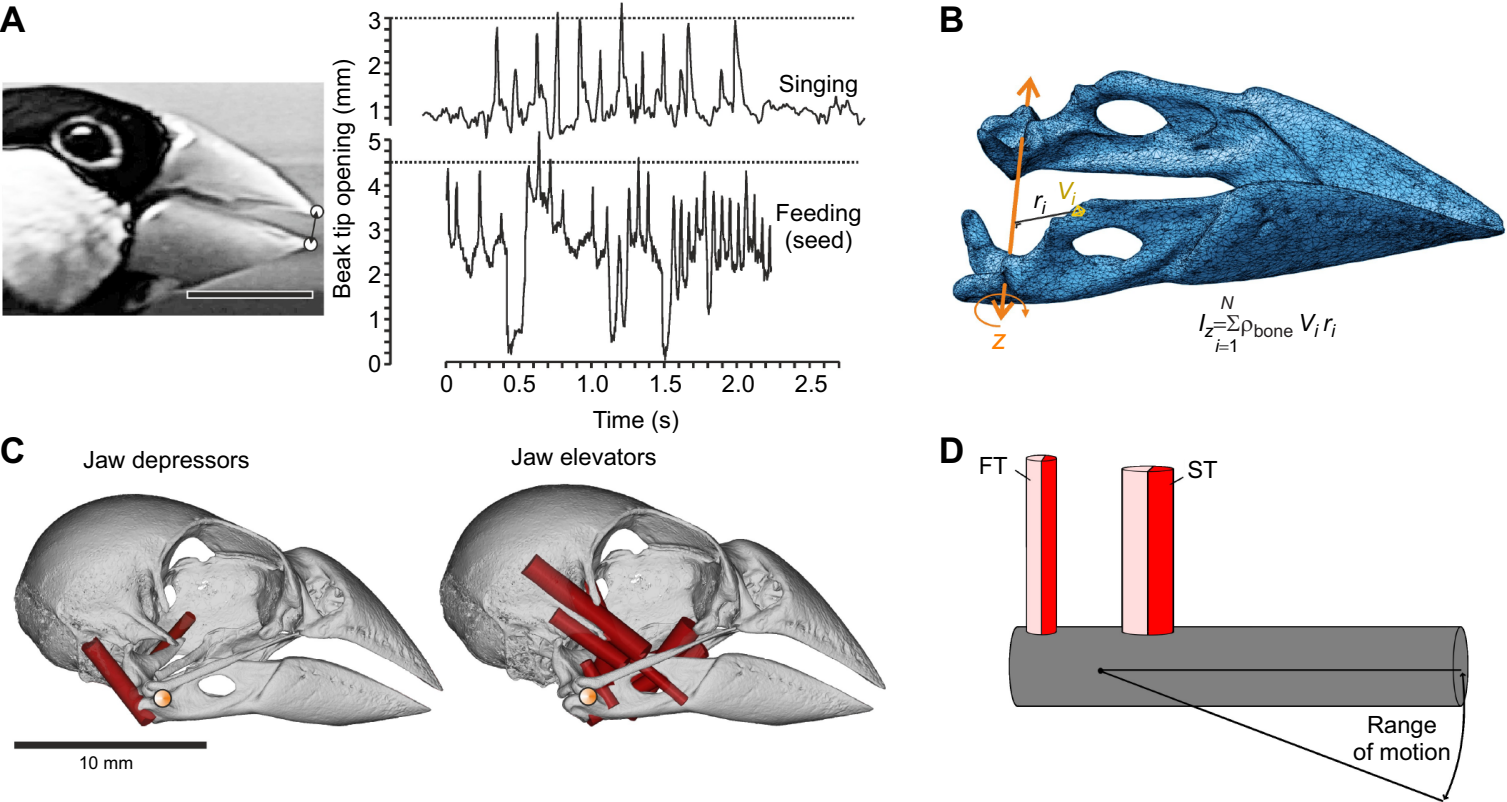
**Model outline parameters.** (A) Beak tip opening kinematics during song and feeding. (B) The mandible of the Java sparrow from which we calculated the inertia around the *z*-axis (*I_z_*). We used the density of the bone (ρ_bone_) from Dumont (2010; 2200 kg m^−3^). *V_i_*, volume of bone element *i*; *r_i_*, distance of bone element *i* to rotational axis. (C) The depressor and elevator muscles we used to calculate the total depressor and elevator force. (D) Schematic of our model. The mandible is represented as the single rod on which two muscles are attached: the depressor and elevator muscle. Both muscles consist of both fast-twitch (FT) and slow-twitch (ST) muscle fibers.

We recorded beak amplitude movement during singing and seed feeding based on 1000 frames s^−1^ high-speed videos (Fastec TS3; RDI Technologies, Knoxville, TN, USA) of an adult male *L. oryzivora*. The bird was held in a 30×50×50 cm cage with *ad libitum* food and water availability. The fragments in which the head was held approximately perpendicular to the lens axis for several seconds were selected. Landmark tracking of the beak tips showed amplitudes of approximately 3 mm during singing and 4 mm during feeding (Fig. 3A). Kinematic profiles can later be compared with the model output. The experiment was approved by the ethical committee animal experimentation of Ghent University (EC2013-069).

As the mandibular is the main rotatory body, we represented this in our model with a rod that has the same length (20.79 mm; Genbrugge et al., 2011) and inertia (3.1947 10^−8^ kg m^–3^) as the mandible (Fig. 3B,D). We calculated the mass by multiplying the volume with the bone density found in birds' skulls (2200 kg m^−3^; Dumont, 2010).

### Forward dynamics model

We constructed our simple model in MATLAB's Simulink (version R2022a, MathWorks, Natick, MA, USA) environment using the Simscape Multibody package. The model consists of a rod with the properties of the Java sparrow’s mandible (see ‘General setup model’). A muscle was placed on each side of the joint, representing all combined adductor or abductor muscles found in the jaw musculature. Using the model, we investigated the frequency of the rod oscillations generated by the muscle contractions and activation patterns. The model was solved using fixed time steps of 0.00001 s with an ode1be (Backward Euler) solver.

### General setup model

As orientations of the beak can vary from horizontal to almost vertical during feeding and singing, we deactivated gravity in the model. The joint simulates the quadrato-mandibular joint and has a restricted range described by the upper beak and the elastic properties of the muscle–tendon unit (MTU), which we set as 45 deg. Before reaching these limits, the movement is damped by a coefficient of 1000 Nm deg^−1^ s^−1^ over a range of 0.1 deg.

To implement the muscle morphology into the model, we used the anatomical data from the literature (Genbrugge et al., 2011). We calculated the weighted average of CSA (1.66×10^−5^ and 2.18×10^−6^ m^2^), muscle length (0.0060 and 0.0063 m) and moment arm (0.0028 and 0.0014 m) of abductors and adductors, respectively (Fig. 3C). To serve as a baseline configuration, we equalized the moment arms of abductor and adductor muscles. The little information available on jaw muscle fiber type in birds shows that a granivorous bird (*Gallus domesticus*) has 50% FT and ST muscle fibers (Dubale and Muralidharan, 1970). Therefore, we virtually divided both abductor and adductor muscles in the model into two muscle bindles: one with FT fibers, and one with ST fibers. These bundles can be activated independently to set recruitment scenarios (see ‘Twitch characteristics’). At the end of each muscle, we added a short, stiff tendon (10% of muscle length).

### The muscle–tendon unit

**Fig. 4.**
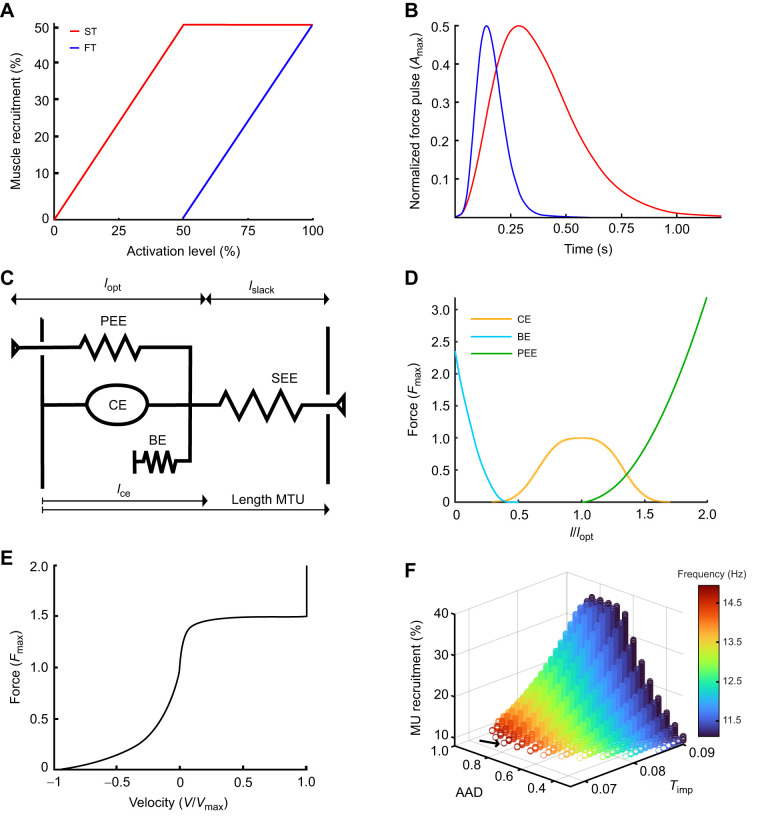
**Illustrations of model parameters and optimization.** (A) Representation of Henneman's principle with ST fibers being recruited before the FT fibers in a muscle composed of 50% ST and 50% FT fibers. (B) Differences between the normalized force pulses of the ST and FT muscle fibers. (C) Muscle tendon model adapted from Geyer and Herr (2010). The muscle consists of a contractile element (CE), a parallel elastic element (PEE) and a buffer element (BE). Together with the serial element (SE), the CE, PEE and BE form the muscle tendon unit (MTU). *l*_ce_, CE length; *l*_opt_, muscle length for highest isometric force output; *l*_slack_, rest length. (D) Force–length relationship used in the simulations. The BE is present as a force to prevent the muscle from collapsing when it becomes too short. (E) The force–velocity relationship used in the simulations. Notice the force rise at a *V*_max_ of 1 to restrain the eccentric velocity to increase beyond *V*_max_. (F) Model optimization results showing the link between frequency (heatmap scale) and the three variable parameters. The model with the highest frequency output is selected for each model configuration, while still fulfilling the amplitude criterium. The model with total highest frequency is indicated with the black arrow in this example. AAD, antagonist activation delay; MU, motor unit; *T*_imp_, time between the start of two successive pulses.

We used the muscle–tendon model from Geyer and colleagues (Geyer and Herr, 2010; Geyer et al., 2003). This Hill-type model consists of four parts: a contractile element (CE), a parallel elastic element (PEE), a buffer element (BE) and a serial elastic element (SEE) (Fig. 4C). The force of the MTU (=*F*_m_=*F*_see_=*F*_ce_+*F*_pee_–*F*_be_) is computed by resolving the inner degree of freedom of *l*_ce_, where *l*_ce_ is the length of the CE and equal to ∫*v*_ce_d*t*=∫[*f*_v_(*v*_ce_)]^–1^d*t*, where *f*_v_(*v*_ce_) is the force–velocity relationship (Fig. 4E). The force of the contractile element is given as *F*_ce_(ACT,*l*_ce_,*v*_ce_)=ACT*F*_max_*f*_l_(*l*_ce_)*f*_v_(*v*_ce_). The generated CE force depends on the muscle's activation (ACT), the isometric maximal force (*F*_max_), the activation state, and the force–velocity and force–length [*f*_l_(*l*_ce_)] relationships (based on Aubert, 1956). This means that *f*_v_(*v*_ce_)=(*F*_see_–*F*_pee_+*F*_be_)/ACT*F*_max_*f*_l_(*l*_ce_). The BE rest length is *l*_min_=*l*_opt_–*w* with a reference compression of ε_BE_=*w*/2. The PEE force is defined as *F*_pee_=*F*_max_[(*l*_ce_–*l*_opt_)*l*_opt_ε_pee_]^2^*f*_v_(*v*_ce_) with the PEE reference strain ε_pee_=*wF*_pee_∼*f*_v_(*v*_ce_), which allows us to rewrite *f*_v_(*v*_ce_)=(*F*_see_+*F*_pee_)/[ACT*F*_max_*f*_l_(*l*_ce_)+*F*_pee_^*^ with *F*_pee_^*^=*F*_max_[(*l*_ce_–*l*_opt_)*l*_opt_ε_pee_]^2^, which can be integrated with coarse steps because the value cannot be negative [*f*_v_(*v*_ce_)<0]. The BE and PEE lie outside the common work range of the muscle and will not be encountered during our simulations.

The force–length and force–velocity relationships are given, respectively, by the next equations:
(1)

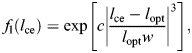
and
(2)

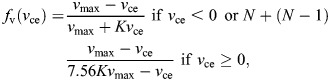
where *l*_opt_ is the length for maximal contractile force production, *w* describes the width of *f*_l_(*l*_ce_) and *c* is the residual force factor of *f*_l_(*l*_ce_). *v*_ce_ is the contractile velocity with *v*_max_ the maximal contractile velocity (described in muscle lengths per second). The *K* factor describes the curve of *f*_v_(*v*_ce_)<0 and *N* is the eccentric force multiplication factor.

The tendons are described as a non-linear SEE: *f*_see_(ε/ε_ref_)^2^ if ε>0 or 0 if ε≤0 with *f*_see_(ε_ref_)=1 and ε=(*l*_see_–*l*_slack_)/*l*_slack_, where *l*_see_ is the length of the SEE and *l*_slack_ is the rest length of the tendon.

### Muscle physiology and anatomy

With the model in place, we conducted a literature study and investigated the results of the feeding/singing Java sparrow to assign the most appropriate values for the muscle parameters.

*l*_opt_ is the length at which the isometric force production is maximal. If the length exceeds this value, the PEE comes into play (Fig. 4D). Generally, common movements are performed on the ascending limb of the force–length curve. Yet, there are examples of jaw muscles working on the descending limb (Gidmark et al., 2013; Taylor et al., 2019). If this were the case in the muscles of the Java sparrow, PEE would presumably be shifted further down the descending limb. For the depressor muscle, we decided to have the *l*_opt_ at the muscle length at which the beak is closed. For the depressor muscle, we chose a muscle length for which the beak-tip is opened for 4.5 mm. As the beak-tip distance during singing is around 3 mm, both muscles will operate on the ascending part of the force–length curve.

The force–length curve is further described by *w* (relative width of the curve) and the residual force factor *c*. The width of the bell curve is described by the length of the sarcomeres (e.g. Lieber and Ward, 2011). Because, among mammals, the myosin filament length is approximately constant (1.6 µm), sarcomere length differences are due to the length of actine, which can differ from 0.95 µm in frogs to 1.3 µm in humans (Walker and Schrodt, 1974). This results in possible muscle contractions of 37% in frogs or 53% in humans. We chose to keep *w* similar to what was used in previous models based on humans (Geyer and Herr, 2010; Winters, 1990). As configured in our model, the muscles will operate on or close to the bell-curve plateau. These settings will therefore have a negligible effect.

The force–velocity curve (Fig. 4E) is described by two factors, *K* and *N*. The constant *K* defines the curvature of the concentric part of the curve. The curvature can differ between ST and FT fibers (Close, 1964), but for simplicity, we decided to retain the original value for both fiber types used in other models (Geyer et al., 2003, 2006). The eccentric force multiplication *N* plateaus at 1.6 to 1.8 of the maximal isometric force in from muscle fibers (Alcazar et al., 2019). As there is little other research performed on this topic, we chose the multiplication factor of 1.5, similar to other models (Geyer et al., 2003; Geyer and Herr, 2010; van Soest and Bobbert, 1993).

As we implemented both ST and FT muscle fibers, we had to decide which factors differed between the muscle fiber types. Both *F*_max_ and *V*_max_ of the fiber types differ substantially. ST muscle fibers produce between 40% and 70% of the FT muscle fiber forces in humans (Linari et al., 2004) and 50% in rat plantaris muscles (Bottinelli et al., 1991). We chose the ST fibers to have 55% of the maximal force of the FT muscles. The value of 25 N cm^−2^ is often chosen as an average of the muscles' force production. However, as these muscles consist of a mixture of both muscle fiber types, we decided that the mean output of both muscles should be 25 N cm^−2^. Therefore, the output of the FT and ST muscle fibers was set at 32.3 and 17.8 N cm^−2^, respectively.

The maximal shortening velocity differs with both fiber types, as the ST is slower than the FT fibers. It also depends on animal size, as in general, smaller animals have larger contractile speeds (Medler, 2002). The contractile speed, described in muscle lengths per second (ML s^−1^), differs strongly in literature and models. ST muscle fibers are 4 to 6 times slower in humans (Linari et al., 2004). In rats and mice, the contractile velocities were calculated to be 6–13 ML s^−1^ for ST and 9–24 ML s^−1^ for FT muscle fibers (Close, 1964; Ranatunga and Thomas, 1990). Hence, as our model species is relatively small, we chose contractile speeds similar to that of mice (Table 1).

**Table 1.** Parameters used in baseline model for both fast-twitch and slow twitch muscles

Parameter	Fast-twitch muscle	Slow-twitch muscle
Activation		
Time to peak (s)	0.015	0.030
Half relaxation time (s)	0.008	0.024
Muscle		
*V*_max_ (*l*_opt_ s^−1^)	18.3	7.1
*F*_ratio_ (*F*_max_)	1	0.55
Muscle fiber proportion (%)	50*	50*
	Elevator muscle	Depressor muscle
*l*_slack_ tendon (m)	6.2×10^−4^	6.2×10^−4^
Moment arm (m)	0.0021*	0.0021
*l*_opt_ (m)	0.0063*	0.0062
*F*_max_ (N)	10.71*	1.41

*Subject to change while changing model parameters.

As we are not interested in the effect of tendons on the output of our model, we decided to have a short (10% of muscle length) tendon. We decided to use half the strain of 0.04, often used in other research (Geyer et al., 2003; Geyer and Herr, 2010; Thelen, 2003). Owing to the short length and low reference strain, tendons in our model will show negligible strain.

### Twitch characteristics

The muscles are activated using single twitches, following the shape of normalized force pulses with a structure adapted from the work of Raikova et al. (2007) (see Fig. 4B). The normalized force pulses consist of five parameters: (1) *T*_ttp_ (time to peak, or the duration between the start of force production and when it reaches *F*_max_), (2) *T*_hr_ (half relaxation time, or the time from the maximal force output to *F*_max_/2), (3) *T*_imp_ (time between the start of two successive pulses), (4) *A*_max_ (maximal activation multiplied by the proportion of MUs activated) and (5) *C* (a small constant because the activation cannot be zero because of the algorithm used for solving the force–velocity relationship requires non-zero forces). The twitch force pulse profiles are described as follows:
(3)

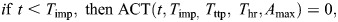


else:
(4)

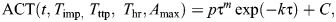
where τ=*t*–*T*_imp_, *k*=ln2/{*T*_hr_–*T*_ttp_ ln[(*T*_hr_+*T*_ttp_)/*T*_ttp_]}, *m*=*kT*_ttp_ and *p*=*A*_max_exp[–*kT*_ttp_(ln*T*_ttp_–1)].

The twitches can overlap, but no summation is made of the overlapping twitches to simplify the simulation. Because the action potential stimulus is not modelled explicitly, no latencies between twitch time and onset of force production are used.

The *T*_ttp_ differs between the muscle fiber types: rat ST muscle fibers have a *T*_ttp_ of 34 ms and FT muscle fibers of 14.5 ms in (Ranatunga and Thomas, 1990), and catfishes have a *T*_ttp_ of 15 ms. Therefore, we chose a FT muscle fiber *T*_ttp_ of 15 ms and ST muscle fiber *T*_ttp_ of 30 ms.

The maximal value that the activation can reach (*A*_max_) is defined by the maximal value of the twitch signal. If an activation of one equals a tetanus contraction, the twitch value has to be lower. The FT muscle fibers in the rat gastrocnemius reach a maximal twitch value of 0.2, but also differ from ST muscle fibers (Grotte and Celichowski, 1993). As we already differentiated the *F*_max_ of the muscle fiber types, we will not differentiate the maximal activation, as this affects the maximal force output.

We chose a fast half-relaxation time (*T*_hr_) that is based on the FT muscle fibers of lizards (Bennett et al., 1984) and the pectorals muscle of the blue-breasted quail (Askew and Marsh, 2001). The *T*_hr_ of the FT fibers was an approximate of two (Askew and Marsh, 1997) and four (Wallinga-de Jonge, 1980) times faster than the ST fibers. Therefore, we decided to have the ST fiber *T*_hr_ that is three times slower than the FT fibers.

The activation patterns for the same muscle were described by the amount of twitches, the duration between the twitches (*T*_imp_), and the amount of recruited MU. As the existing model is of an antagonistic muscle pair that undulates the lever, the time point at which the antagonist is activated needs to be determined. To do this, we introduced the antagonistic activation delay (AAD) term, which shows when the antagonist is activated between two twitches of the agonist. This is a value between 0 and 1, with 0 meaning that the twitch starts with the first twitch of the agonist and 1 with the following agonist twitch. In addition, both ST and FT muscle fibers are recruited following the orderly recruitment of MU (Henneman's principle; Fig. 4A). Depending on the quantity of the muscle fiber type, only ST or a combination of both ST and FT were activated. We decided that the normalized force pulses follow twitch activation patterns, which results in a maximal 20%, equal to *A*_max_, of the maximal force output. The recruited MU are in relation to a maximal twitch output, meaning that 50% motor unit recruitment (half of *A*_max_) corresponds to 10% maximal isometric force production.

### Muscle model parameters and lever motion targets

In Table 1, the starting input parameters of the model are shown. Three variable parameter inputs are used to find the output with the highest frequency output of lever oscillations: *T*_imp_, AAD and recruited MU. There are two constraints in the model: the lever has to rotate so that the center of the lever's tip has translated 3 mm, and the lever tip has to go back to its original position afterwards (with a tolerance of 0.5 deg). This movement is performed four times to rule out start-up effects. First, the elevator muscle is activated, but as the lever is already at the ‘fully elevated’ position, this leads to an isometric muscle contraction.

### Model optimization

To find the optimal baseline model output, we ran the model multiple times while changing the following three optimization parameters: *T*_imp_, AAD and MU. We altered AAD ranging from 0.1 to 0.9 in steps of 0.05, the MU between 1% and 36% in steps of 0.1% and *T*_imp_ from 0.01 to 0.09 s in steps of 0.01 s (Fig. 4F). To investigate the effect of the adaptations for increased static torque, discussed in the Conceptual framework section (mass, moment arm, etc., referred to as torque parameters), we started by making the smallest torque parameter change (for example, mass increase of 1.5 times) and searched for the optimal frequency by changing the three optimization parameters. We did this similarly by changing *T*_imp_, AAD and MU, in intervals described above, but now starting from the parameter values of the optimal baseline model. Once we found the new optimal frequency output of the first torque parameter change, we repeated the steps but now started from the optimal output of the previous torque parameter change. The specific values of the optimization parameters for each torque parameter are shown and Table S1.

## RESULTS

### Optimization of activation pattern in the baseline model

The baseline model has a larger CSA of the elevator muscle, which has a combination of both FT and ST muscle fibers. Still, unlike the bird it is modeled after (Fig. 3C), it has equal moment arms for elevators and depressors. With a high amount of elevator MU activated, the lever fails to depress enough to reach the target lever tip excursion of 3 mm. The model solution that fulfils the imposed constraints is reached when the MU recruitment of the elevator muscle is higher than 9% and is optimal at 13.0%. Decreasing the MU recruitment from 100% gradually results in a decrease in time duration in which the lever is stationary at the upper limit (0 deg) position until it has no stationary time period at 13.0%. In Fig. S1, we show two examples that exhibit mechanical differences between two different strategies (few versus many MUs recruited) to reach similar suboptimal frequencies (12.5 Hz). Interestingly, the recruitment of 13% MU of the elevator muscle is a similar solution to what we hypothesized in the CSA part of the conceptual framework. As the elevator muscle is approximately 7.7 times stronger than its antagonist, the elevator muscle only needs to be recruited for 13% to achieve a theoretical balance.

**Fig. 5.**
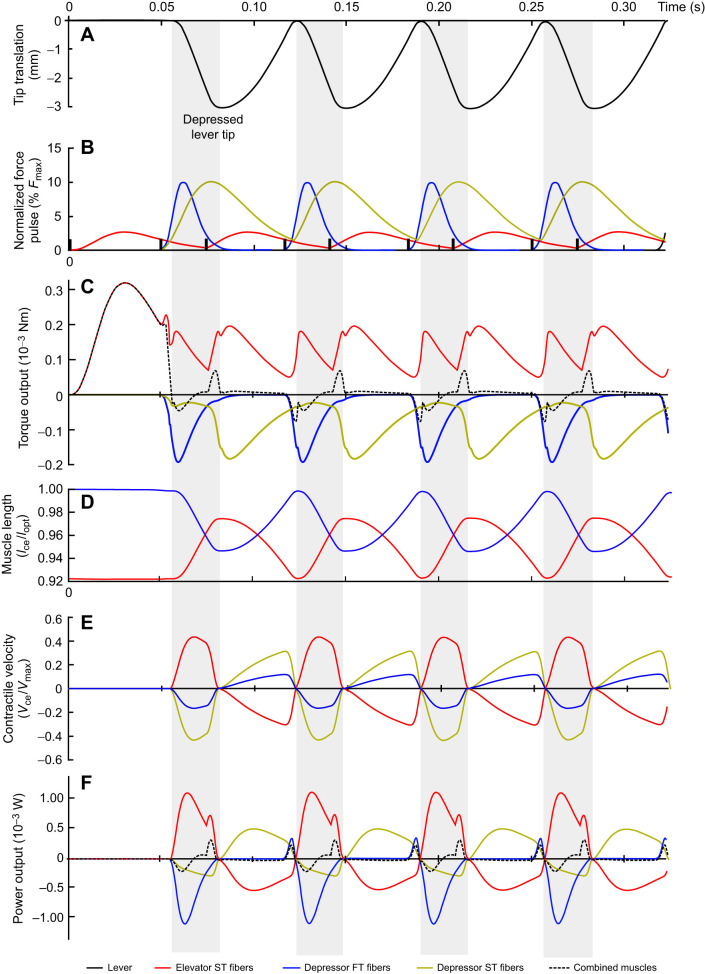
**Output of the baseline model.** (A) The lever tip *y*-axis translation. Gray bars show the depressor phase of the lever. (B) The corresponding normalized force pulse production in relation to maximal force (*F*_max_). This is defined as the activated motor units multiplied by the maximal activation. The vertical black lines show twitch onsets. (C) The torque output of the three activated muscles, and the summed torque. (D) The muscle length of the depressor and elevator muscles. (E) The contractile velocity of the different muscles. (F) The power output of the three different muscles and the summed power of the three combined muscles.

The elevator MU recruitment in the baseline model is lower than 50%. This means that, following Henneman's principle (Fig. 4A), all fibers recruited were of the ST variant. A constant in all the different models is that the depressor muscle is fully recruited (Fig. 5B), meaning that both ST and FT fibers are activated in concert. This implies a velocity difference between the depression and elevation phases of the movement, with a relatively fast depression, driven by FT fibers, and a slower elevation owing to the ST fibers (Fig. 5A). This is similar to what we hypothesized in the conceptual framework (Fig. 2). As both fiber types have different force rise and relaxation speeds (Table 1), the torque peaks of the ST and FT fibers of the same muscle do not coincide. As the dominant force pulses to reach the highest frequencies are those of the depressor FT fibers and elevator ST fibers, the least antagonistic normalized force pulse overlap is reached when the depressor is activated at 70% to 75% of the activation cycle. The overlap is minimized, but opposing torque overlap is still present (Fig. 5C), certainly because both antagonistic muscles are always activated owing to the slow relaxation time not being able to reduce the normalized force pulse to zero before the next pulse starts.

**Fig. 6.**
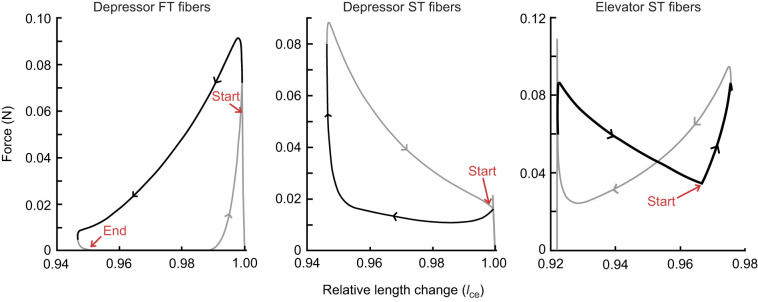
**Work loops of the baseline model.** Work loops from the three activated muscles, in which the dark lines indicate the phase of lever depression, and the grey lines the phase of lever elevation. The red ‘start’ arrow indicates when the muscle's force pulse starts, and the red ‘end’ arrow indicates when the pulse stops. As both the ST elevator and ST depressor muscles do not relax to a zero-force state before the next twitch pulse starts, only ‘start’ arrows are shown.

**Fig. 7.**
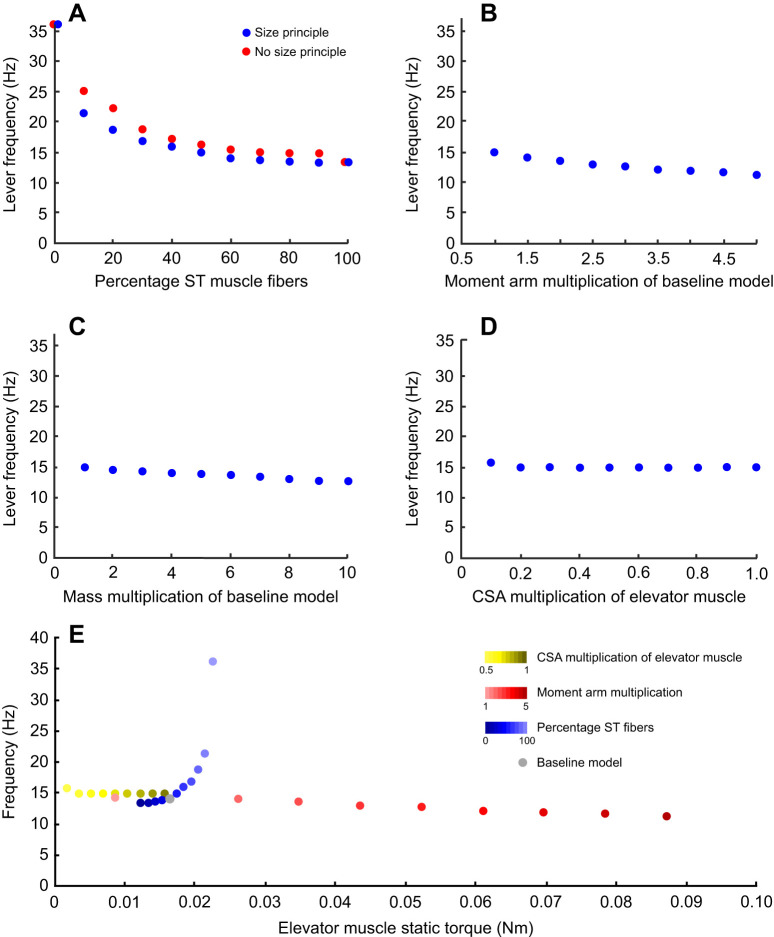
**Sensitivity analysis of peak lever frequency.** (A) The effect of fiber type is tested with and without including the size principle (Henneman's effect). (B) Effect of changes in moment arm, (C) lever mass and (D) elevator CSA. (E) the frequency output compared to the static torque capabilities of a changed moment arm, CSA and slow fiber type percentage. They grey dot represents the baseline model.

ST fibers of both the depressor and elevator muscles perform either a full or partial damping effect on the lever movement (Figs 5C,F and 6). The ST depressor muscle contributes little to the depressing phase but then plays a prominent role in reducing the net elevating torque, which results in a net negative work output (Fig. 7). Deactivating this muscle would result in a model output with a frequency of 21.74 Hz while only activating 4.5% of the elevator muscles. The ST elevator muscle has both a motor and a damper phase, elevating the lever but also resisting the movement to the depressed position. Increasing the MU of the elevator muscle has a dual effect. Increasing the available elevator torque but also diminishing the net depressor torque output, thus reducing the depressing velocity of the lever.

When the model starts optimizing for maximal frequency output, it soon results in antagonist co-contraction between the ST muscle fibers (Fig. 5B). Although the target frequency increases during the optimization trials, the magnitude of co-contraction eventually becomes so high that the necessary lever tip amplitude can no longer be reached. The model has then just passed the upper frequency limit of the task. Interestingly, this implies that the twitch activation profile (*T*_ttp_, *T*_hr_) will largely dictate the *T*_imp_ value, in combination with AAD, and therefore the highest frequency. Consequently, changing the activation profile of the ST fibers will shift the optimal frequency output but will not fundamentally change the model's behavior.

### Effect of mass

To investigate the effect of mass, we increased the starting mass 10 times in nine gradual steps. There was a linear reduction in frequency with increasing mass, starting from 14.93 Hz at the Java sparrow beak mass to 12.69 Hz at 10 times the mass (Fig. 7C, Table S1). The only substantial difference in muscle function after optimization was an increased time between the force pulses (*T*_imp_), which resulted in the lever movement's frequency decrease. The recruited elevator muscle fibers (MU) remained close to 13%. With an increase in inertia, it takes longer to depress the lever to the required position, requiring longer periods between the normalized force pulses. If the elevator muscle responded to the increased mass by recruiting larger amounts of elevator MU elevate the lever faster, it would also reduce the time the lever can depress because of torque overlap. This would result in a lever that cannot reach the target depression angle unless the *T*_imp_ increases, and hence frequency would decrease even further. This is in accordance with what we described in the conceptual framework (Fig. 1). A small decrease of AAD from 0.7 to 0.65, from 1× mass to 10×, occurred when the frequency decreases, meaning that the antagonist pulse sequences become slightly closer to the symmetrical situation (AAD=0.5). This is a general effect of an increase in *T*_imp_.

### Effect of moment arm elevator muscle

We increased the moment arm of only the elevator muscle up to 5 times in steps of 0.5. With changing the elevator muscle moment arm, we increased the *l*_opt_ so that it would remain at a point where the lever tip was depressed for 4.5 mm. This means that the relative *l*_opt_ always remains lower than 1. An increase of the moment arms 5 times the original length resulted in a frequency decrease of approximately 25% (from 14.93 to 11.21 Hz; Fig. 7B; Table S1). With this increase in the moment arm, the recruited MU decreased from 13.0% to 6%.

The drop in frequency is due to two factors. First, a longer elevator *l*_opt_ did not increase *V*_max_ sufficiently to cope with the additional distance the muscle has to contract to produce the required lever movements. As the moment arm increases 5 times, the displacement of the elevator muscle increases by a similar amount, but the velocity increases only 3.3 times. Hence, the elevator movement takes longer to complete, decreasing the frequency. Second, by changing the moment arm, the stretching velocity of the elevator muscle during the depressor phase increases similar to the moment arm increase. Starting from a moment arm of 2, the stretching velocity reaches a relative *V*_max_ of 1. As shown in Fig. 7B, at a *V*_max_ of 1, a resisting force is modelled, restraining the *V*_max_ to this value. Hence, the depressing phase is limited by the *V*_max_ of the ST elevator muscle fibers, restraining the maximal depressing velocity of the lever. In conclusion, both the elevating and depressing phases increase in time, meaning the frequency decreases.

### Effect of muscle fiber type

The effect of muscle fiber type is substantial on the frequency output of the model. The model with only fast muscle fibers has a frequency output of 36.1 Hz and decreases to 13.3 Hz in the model with only ST muscle fibers (Fig. 7A). While changing the amount of FT fibers from 0% to 100%, the recruited MU shifts between 9.6% and 13.0%. The frequency decrease is not linear, as the largest frequency shift happens while changing the amount of ST fibers from 0% to 10% (decrease from 36.1 to 21.4 Hz; Table S1). Owing to the implementation of the size principle, with a shift from 0% to 10% ST fibers, all the recruited fibers of the elevator muscle were ST. As a result, the lever closing speed is reduced by a factor of two (14 to 28 ms), which is in accordance with contractile velocity difference between FT and ST fibers (Table 1). In addition, the depressor phase of the lever also increases due to two factors. First, a reduction of FT muscle fibers by 10% results in a reduction of the depression speed due to a reduction in contractile speed of the entire muscle. Second, the depressor's ST fibers start damping the elevation due to eccentric contraction (Fig. 5C). As the proportion of ST muscle fibers increases, the two aforementioned effects increase. Although there are large effects by the shift from 0% to 50% ST fibers, the differences in frequency from shifting from 50% to 100% ST fibers become rather small (14.93 to 13.30 Hz).

### Effect of orderly recruitment

To test how the orderly recruitment of muscle fibers influences the frequency output of the lever, we compared the baseline model with one in which both depressor and elevator muscles recruit, if possible, equal amounts of ST and FT muscle fibers. We investigated the influence while changing the amount of FT muscle fibers starting from 0% to 100% in steps of 10% (Fig. 7A). Evidently, the extreme frequencies are similar to that of the models with orderly recruitment, as they consist of only slow or fast muscle fibers. The model with no orderly recruitment follows the same trend as the model with orderly recruitment while changing the muscle fiber proportions, although in general, the frequency is 10% to 15% higher. Interestingly, although the duration of the depression phase of the lever changes more or less linearly with an increase in the proportion of fast muscle fiber type, this is not the case for the elevation phase. The duration increases proportionally with the opening phase duration from 0% to 60% FT fibers. Beyond 60% ST muscle fibers, the closing phase duration decreases, and from that point, the frequency stays similar.

### Effect of muscle force imbalance

Lever system balance can be reduced or restored by reducing the CSA of the elevator muscle. To investigate the effect of the potential force difference between antagonistic muscles, we decreased the CSA of the elevator muscle in steps of 0.1 of the CSA_,_ until the CSA of the antagonists were equal. The frequency output changed from 14.93 to 15.63 Hz with equal antagonistic muscle *F*_max_ (Fig. 7D). The frequency only changed when the CSA of antagonists were equal, meaning that the frequency output stayed similar for all changes in CSA in between. As described in the conceptual framework, an increase in MU recruitment (from 13% to 100% over the CSA range) of the elevator muscle counters the decrease in CSA.

## DISCUSSION

Our analysis clearly shows that the torque–frequency trade-off is a special type of force–velocity trade-off. The successions of back-and-forth motions strongly influence the musculoskeletal dynamics and, hence, the way modifications of this system may constrain its performance. In contrast to single start–stop movements such as a closing jaw (Van Wassenbergh et al., 2005), a striking appendage (e.g. Patek et al., 2004), vertical jumping (e.g. Aerts, 1998) or a kicking leg (e.g. Kellis and Katis, 2007), muscle relaxation rate becomes essential, and effects of imbalance between agonist and antagonist come into play (Fig. 5). This shows the importance of explicitly referring to the cyclicity of the movement (i.e. performance in terms of frequency) when discussing a potential trade-off on this kind of movement.

The case study we analyzed in the model simulation part, namely the cyclical movement of the lower beak of the Java sparrow, pointed to some noteworthy effects. Our sensitivity analysis shows that fiber type has the most substantial impact of all variables tested (Fig. 7A). This could be expected given the examples of high-frequency systems in which super-fast muscles evolved (Elemans et al., 2008, 2011; Young and Rome, 2001; Rome and Lindstedt, 1998). Still, for a multi-functional system in which mixtures of FT and ST fibers occur, we showed that a substantial drop in frequency output could already happen with only 10% ST fibers versus a muscle with only FT fibers. The effect of fiber-type composition in muscle levels off when the ST fibers become dominant (Fig. 7A). For bird beaks in particular, including small MUs with ST fiber could be important for their ability to perform precise, low-force manipulation of items. As ST types are less prone to fatigue, this would also imply improved endurance for such tasks. Consequently, low-force precision handling and endurance are expected to be strongly traded against adaptations for increased static torque.

We showed that Henneman's effect (orderly recruitment) could significantly reduce the frequency output of muscle systems with mixed fiber types. Henneman's principle states that MUs are activated hierarchically based on the firing rate, with fast-firing MUs activating before the slower-firing ones (e.g. Azevedo et al., 2020; de Luca and Contessa, 2012; Henneman, 1985), which coincides with predominant activation of ST fibers before FT fibers (e.g Desmedt and Godaux, 1977). This phenomenon enables an animal to perform accurate movements using the ST fibers during low muscle activation, which it can maintain for a long duration. In our sensitivity analysis, this effect reduced the maximal frequency by 10–15% compared with when both fiber types were always activated together. Note that the torque imbalance between the antagonists is an important factor in this process, as this causes the low-level activation of the hypertrophied elevator (i.e. bird jaw adductor), and hence predominantly ST fibers being active (Fig. 2).

Our model indeed pointed to a strongly reduced level of activation of the relatively large jaw adductors of the Java sparrow to achieve high frequencies of beak movement. This model, however, had a high resolution in selecting the activation level, as if there were 1000 MUs. Animals, however, are limited in their force-production resolution by the number and size of the MUs of their muscles (Duchateau and Enoka, 2022). This implies that the birds may not be capable of achieving the theoretical maximum of the model, and either overactivate or underactivate the muscle. If the jaw adductors were overactivated, one might expect short pauses after the closing of the beak. This situation indeed seems to occur judging from the presented Java sparrow beak kinematics (Fig. 3A). Data on MUs, their recruitment and the effect of beak precision would be highly valuable to gain insight into how beak motion is regulated.

As the modeled musculoskeletal lever can be seen as a system with relatively low inertia and without external forces, we initially expected speed and frequency to be Hill-limited (Labonte, 2023). Calculating the effective inertia (following Labonte, 2023) gives a value of 3×10^−5^, indicating that this should indeed be the case. However, in the baseline model, the elevator contractile speeds only reached a maximum of 1/3 *V*_max_ (Fig. 5E) indicating that the muscle operates at its maximal power output. Hence, another force is present that reduces the relative *V*_max_ of the muscles. A ‘parasitic force’ comes from the antagonist muscle: during positive torque output of the depressor muscle, only 8% of this muscle's torque is used to generate movement; the other 92% is countering the antagonist. The same pattern is visible for the elevator muscle, as only 19% is used to generate movement. In comparison, pigeons only generate around 15% negative work for both upstroke and downstroke during flight (Biewener, 2011). Muscle activation overlap during high-frequency movements is logical, but the presence of ST muscle fibers increases the overlap between the antagonist activation drastically. Indeed, our model of the depressor muscle with exclusively FT fibers has 40% torque available for movement production, and models with only ST fibers have 14%. Having ST fibers clearly negatively impacts the frequency-generating capabilities of these musculoskeletal multifunctional lever systems, enhancing the torque–frequency trade-off.

The functional restraint of ST muscle fibers on rapid cyclic movements is a known concept. Therefore, it has been hypothesized that alternative recruitment methods to Henneman's principle exist (Blake and Wakeling, 2014; Smith et al., 1980; Wakeling and Horn, 2009; Wakeling et al., 2006), which can be better tuned to the mechanical demands (for a review, see Wakeling et al., 2011). Holt et al. (2014) found that in the rat plantaris muscle, the maximum shortening velocity was higher and the force rise time, relative to *F*_max_, was longer when both FT and ST fibers were recruited simultaneously compared with one fiber type separately. For movements occurring at lower frequencies and requiring longer, tetanic activations compared with the twitches used in our model, earlier deactivation of the ST fibers will help to prevent ST fibers from impeding the next cycle (Blake and Wakeling, 2014). Alternatively, activation can shift to muscles with more suitable fiber-type compositions. During high-frequency paw movements, the cat predominantly activates the fast-fibred lateral gastrocnemius, leaving the slow-fibred soleus muscle inactive (Smith et al., 1980). For high-frequency movements of bird beaks, however, it is currently unknown whether one of these scenarios is used to circumvent the limits imposed by the slow rise and relaxation of ST fiber on maximal movement frequency.

In contrast to the dominant effect of fiber type, increases in muscle moment arm and lever inertia, both adaptations to larger static torque and force production, only minimally influence the maximal frequency in our Java sparrow model (Fig. 7). These results have implications for a classic example of a torque–frequency trade-off: the beaks of hard-biting Darwin's finches such as *Geospiza magnirostris* are assumed to be limited in their movement frequency during singing compared with those of slender-beaked, low bite force species such as *Certhidea olivacea* (Herrel et al., 2009; Podos, 2001). As the recorded difference in syllable rate (or trill rate) between these species, and hence presumably also the beak frequency, is over fourfold (3 Hz versus 13 Hz; Podos, 2001), our model shows that effects of this order are unlikely to be caused by changes in beak mass or muscle moment arm, but rather by muscle mechanics in conjunction with unbalanced antagonistic muscle pairs.

The outcome of our model can have important implications for behaviors that rely on cyclical, low-inertia movements. Despite their low inertia, our model showed that performing such movements near the theoretically maximum frequencies can incur a high energy cost due to antagonistic muscle co-contractions. This adds to the previously noted trade-off between frequency and endurance because of the different twitch kinetics of fast (FT) versus slow (ST) fatiguable fibers. It is therefore not unlikely that certain species display submaximal performance due to these mechanical drawbacks, for example, in quick beak movement during singing or feeding in birds. Still, the output frequency at which the co-contraction overlap becomes proportionally less unfavorable would still be determined by the same properties responsible for the theoretical maxima, namely, fiber type distribution and its activation properties. Hence, our findings would not only be relevant for the theoretical limits of performance, but also have implications for the strategies animals adopt to balance speed and energetic efficiency.

### Conclusions

Our simplified musculoskeletal model representing a bird beak showed that the trade-off between static torque production and the maximal movement frequency is affected by several factors, of which muscle fiber type is the dominant effector. We showed that unbalanced torque-producing musculoskeletal lever systems suffer greatly from the presence of ST muscle fibers. Owing to torque imbalance, the strong muscle will only be activated partially and, therefore, following the size principle will only recruit ST muscle fibers. Long relaxation times of these muscle fibers will result in large overlap between the antagonist force profiles, resulting in large parasitic forces that inhibit fast muscle contractions and reduce frequency output. Therefore, both torque and frequency are also in trade-off with precision handling (slow, low-force movement by ST fibers) and endurance (fatigue resistance of ST fibers). Motor control resolution for high-frequency movement will likely reduce for the enlarged muscle, potentially leading to static phases in the beak kinematics of singing of feeding birds. As mass and moment arm only slightly influence the maximal frequency, these factors are less important for the torque–frequency trade-off.

## Supplementary Material

10.1242/jexbio.250733_sup1 Supplementary information
