# Supplementary material for: A modelling perspective on torque–frequency trade-offs in multifunctional lever systems driven by antagonist muscle pairs
Source: J Exp Biol. 2026 Jan 30;229(2):jeb250733. doi: 10.1242/jeb.250733 (PMC12891941; doi:10.1242/jeb.250733)
Supplement: Supplementary information [file jexbio-229-250733-s1.pdf]

Table S1. model output with highest frequency output. The table parts with white background indicate starting lever and muscle characteristics of which model. The bold letters indicate which parameter is changed to investigate its effect. The multiplications (mult.) of the lever and muscle parameters are in relation to the baseline model. The light gray part of the table indicate the optimal 'variable parameters' to reach the highest frequency (dark gray).

Lever mass mult.	Momen tarm mult.	Slow muscle fiber percentage	Orderly recruitment	Lopt elevator	Elevator Force mult.	T_{imp}	AAD (ratio)	MU recruitment (%)	Frequency (Hz)
1	1	0.5	1	0.0063	**1**	0.067	0.7	13.0	14.93
1	1	0.5	1	0.0063	**0.9**	0.067	0.7	14.4	14.93
1	1	0.5	1	0.0063	**0.8**	0.067	0.75	16.9	14.93
1	1	0.5	1	0.0063	**0.7**	0.067	0.75	19.2	14.93
1	1	0.5	1	0.0063	**0.6**	0.067	0.7	21.2	14.93
1	1	0.5	1	0.0063	**0.5**	0.067	0.75	26.3	14.87
1	1	0.5	1	0.0063	**0.4**	0.067	0.7	31.0	14.93
1	1	0.5	1	0.0063	**0.3**	0.067	0.75	41.9	14.94
1	1	0.5	1	0.0063	**0.2**	0.067	0.7	65.5	14.92
1	1	0.5	1	0.0063	**0.1**	0.064	0.45	100.0	15.63
1	1	**0**	**0**	0.0063	1	0.028	0.55	13.2	35.95
1	1	**0.1**	**0**	0.0063	1	0.040	0.6	10.1	25.04
1	1	**0.2**	**0**	0.0063	1	0.045	0.55	10.8	22.24
1	1	**0.3**	**0**	0.0063	1	0.053	0.6	13.4	18.74
1	1	**0.4**	**0**	0.0063	1	0.058	0.6	14.7	17.16
1	1	**0.5**	**0**	0.0063	1	0.062	0.55	14.3	16.22
1	1	**0.6**	**0**	0.0063	1	0.065	0.5	14.1	15.38
1	1	**0.7**	**0**	0.0063	1	0.067	0.45	14.0	14.83
1	1	**0.8**	**0**	0.0063	1	0.067	0.35	12.9	14.84
1	1	**0.9**	**0**	0.0063	1	0.068	0.3	12.9	14.80
1	1	**1**	**0**	0.0063	1	0.075	0.45	12.9	13.40

Journal of Experimental Biology • Supplementary information

Lever mass mult.	Moment arm mult.	Slow muscle fiber percentage	Orderly recruitment	L_{opt} elevator	Elevator Force mult.	T_{imp}	AAD (ratio)	MU recruitment (%)	Frequency (Hz)
**1**	1	0.5	1	0.0063	§t	0.067	0.7	13.0	14.93
**2**	1	0.5	1	0.0063	1	0.069	0.75	13.8	14.58
**3**	1	0.5	1	0.0063	1	0.070	0.7	13.6	14.27
**4**	1	0.5	1	0.0063	1	0.071	0.7	13.3	14.04
**5**	1	0.5	1	0.0063	1	0.072	0.7	13.0	13.92
**6**	1	0.5	1	0.0063	1	0.073	0.65	12.8	13.69
**7**	1	0.5	1	0.0063	1	0.075	0.65	12.9	13.44
**8**	1	0.5	1	0.0063	1	0.077	0.65	13.8	13.08
**9**	1	0.5	1	0.0063	1	0.078	0.65	13.9	12.83
**10**	1	0.5	1	0.0063	1	0.079	0.65	13.9	12.69
1	**0.5**	0.5	1	**0.0060**	1	0.070	0.45	18.0	14.29
1	**1**	0.5	1	**0.0063**	1	0.067	0.7	13.0	14.93
1	**1.5**	0.5	1	**0.0065**	1	0.071	0.75	10.9	14.12
1	**2**	0.5	1	**0.0068**	1	0.074	0.75	9.5	13.52
1	**2.5**	0.5	1	**0.0070**	1	0.077	0.7	8.5	12.95
1	**3**	0.5	1	**0.0073**	1	0.079	0.7	7.7	12.62
1	**3.5**	0.5	1	**0.0075**	1	0.082	0.65	6.9	12.14
1	**4**	0.5	1	**0.0077**	1	0.084	0.65	6.5	11.91
1	**4.5**	0.5	1	**0.0080**	1	0.086	0.65	6.1	11.66
1	**5**	0.5	1	**0.0082**	1	0.089	0.6	5.9	11.21
1	1	**0**	1	0.0063	1	0.028	0.55	13.0	36.09
1	1	**0.1**	1	0.0063	1	0.047	0.8	9.6	21.36
1	1	**0.2**	1	0.0063	1	0.054	0.85	10.9	18.64
1	1	**0.3**	1	0.0063	1	0.059	0.8	12.6	16.81
1	1	**0.4**	1	0.0063	1	0.063	0.8	13.0	15.93
1	1	**0.5**	1	0.0063	1	0.067	0.7	13.0	14.93
1	1	**0.6**	1	0.0063	1	0.072	0.6	12.5	13.97
1	1	**0.7**	1	0.0063	1	0.073	0.6	12.5	13.73
1	1	**0.8**	1	0.0063	1	0.074	0.55	12.5	13.49
1	1	**0.9**	1	0.0063	1	0.075	0.5	12.9	13.30
1	1	**1**	1	0.0063	1	0.075	0.45	12.9	13.33

Journal of Experimental Biology • Supplementary information

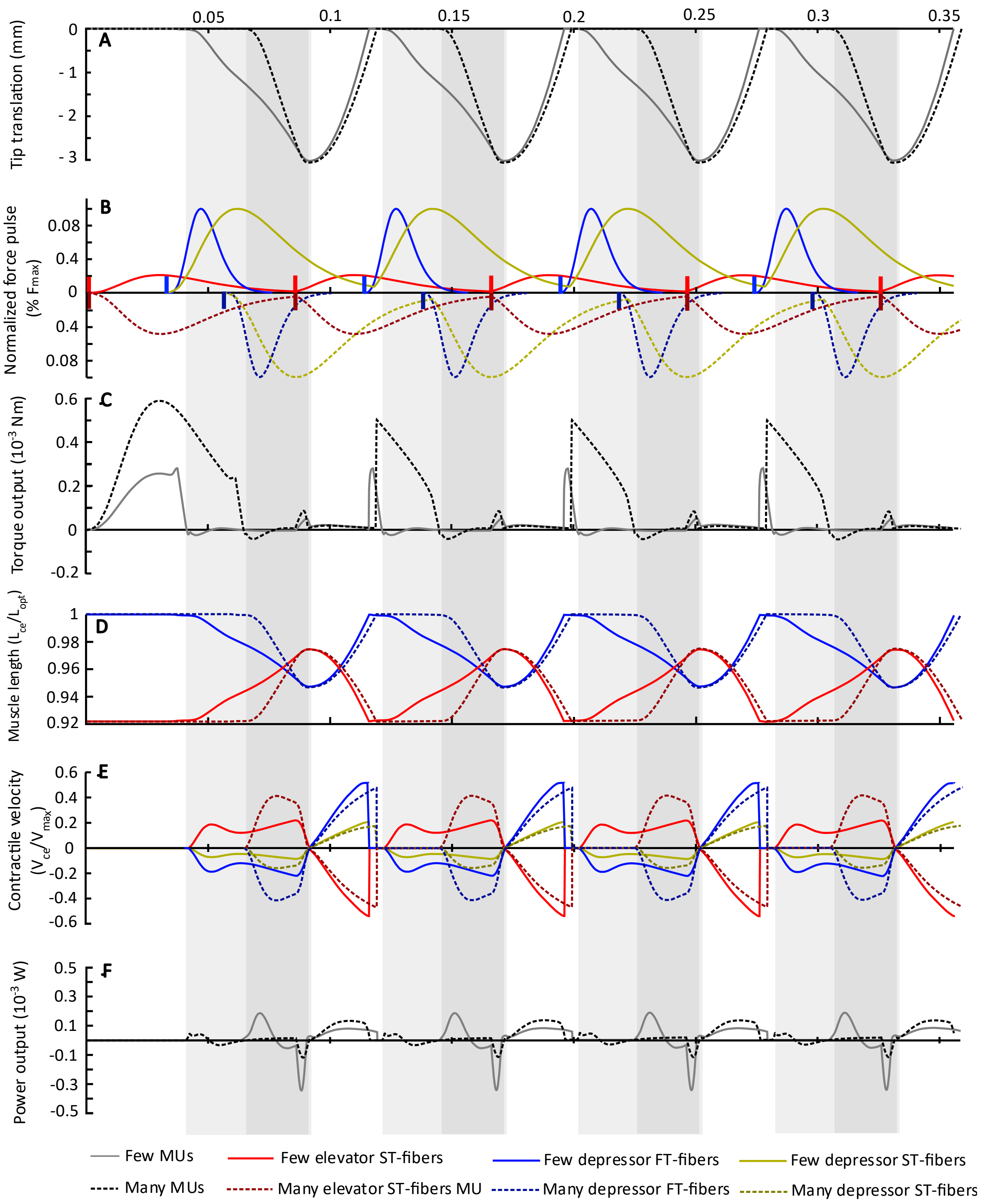

Fig. S1. two different strategies to achieve a suboptimal frequency of 12.5 Hz in the baseline model. One strategy aims to reduce overlap between ST elevator and FT depressor torques while recruiting relatively large amounts of MUs (referred to as 'many MUs model'). This model is similar to the highest frequency output of the baseline model, only with a large period where the lever is stuck in the elevated position. The other strategy aims to reduce overlap between the ST elevator and the ST depressor torques, while recruiting relatively small amounts of MUs (referred to as 'few MUs model'). By doing this, the lever tip's depression speed decreases while the elevation phase stays similar to that of the other model. **A)** The lever tip y-axis translation. Light Grey bars show the depressor phase of lever in the small MU model, the darker grey bar show the depressor phase of the large MU model. **B)** The corresponding normalized force pulse production in relation to maximal force (F_{max}). This is defined as the activated motor units multiplied by the maximal activation. The vertical lines show when the action potentials reach the muscle. The activation pattern of the large MU model are shown underneath the x-axis for clarity, although these variables are also positive. **C)** The torque output of the two different models. **D)** The muscle length of the depressor and elevator muscles. **E)** The contractile velocity of the different muscles. **F)** The power output of the two different model.

Journal of Experimental Biology • Supplementary information
